# Supplementary material for: A G-protein pathway determines grain size in rice
Source: Nat Commun. 2018 Feb 27;9:851. doi: 10.1038/s41467-018-03141-y (PMC5829277; doi:10.1038/s41467-018-03141-y)
Supplement: Supplementary file 1 — Supplementary Information [file 41467_2018_3141_MOESM1_ESM.pdf]

Title:

A G-protein pathway determines grain size in rice

Authors:

Shengyuan Sun, Lei Wang, Hailiang Mao, Lin Shao, Xianghua Li, Jinghua Xiao, Yidan Ouyang*, Qifa Zhang*

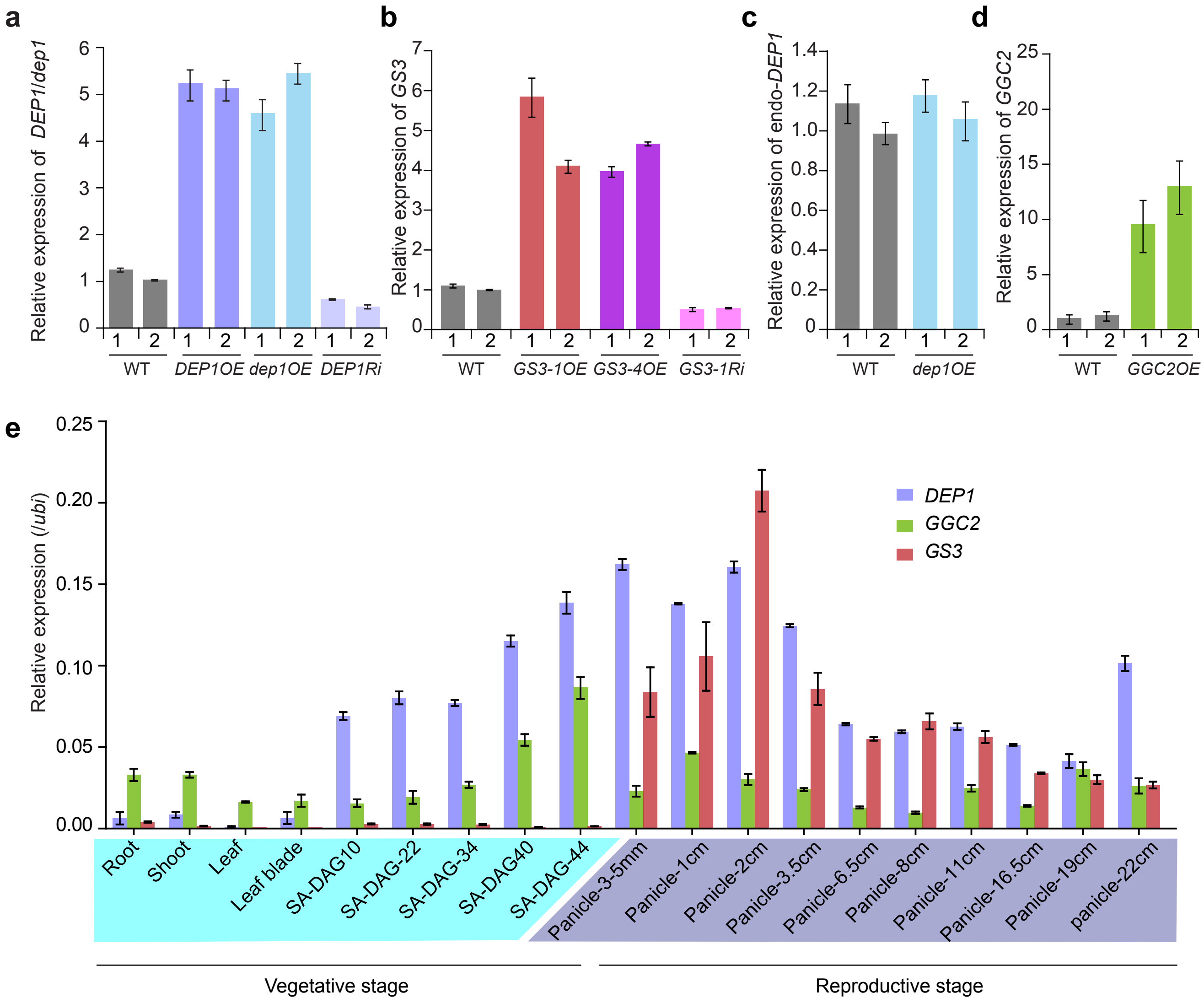

Supplementary Figure 1 Relative expression levels of three Gγ proteins.

(a) Relative expression level of *DEP1* or *dep1* in WT (wild type ZH11), *DEP1OE*, *dep1OE*, and *DEP1Ri* transgenic plants. Transformants from two independent T_2 homozygous lines were assayed for each transgene. Transcriptional level is determined by quantitative Real-time PCR based on cDNA template prepared from 0.5 cm young panicles. Rice *ubiquitin* is used as the reference gene.
(b) Relative expression level of *GS3* in WT, *GS3-1OE*, *GS3-4OE*, and *GS3-1Ri* transgenic plants.
(c) Relative expression level of endogenous *DEP1* in *dep1OE* transgenic plants.
(d) Relative expression level of *GGC2* in *GGC2OE* transgenic plants.
(e) Relative expression levels of *DEP1*, *GGC2*, and *GS3* in root, shoot, flag leaf, leaf blade, shoot apex at 10, 22, 34, 40, and 44 days after germination, and developing panicles in ZH11. SA, shoot apex; DAG, days after germination. Rice ubiquitin is used as the reference gene. Data for all the assays are shown as mean ± SEM (n = 3).

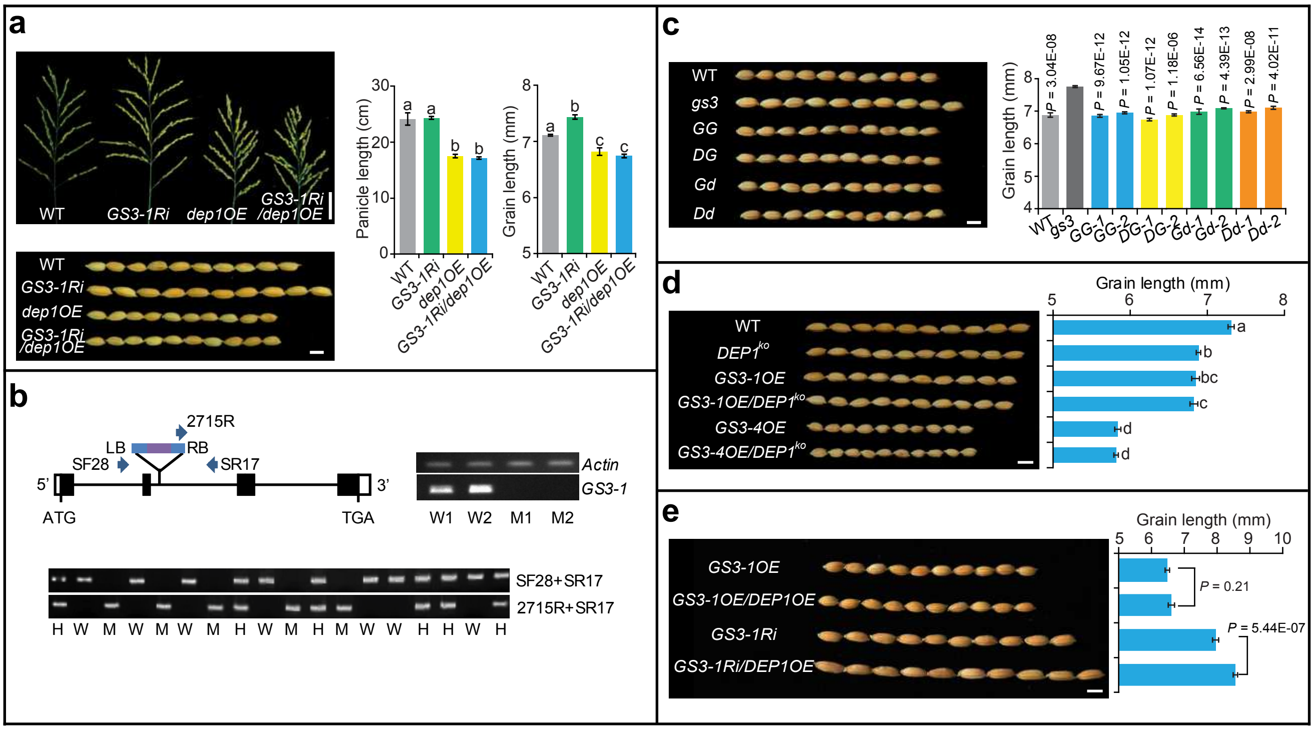

Supplementary Figure 2 Genetic analysis of *DEP1* and *GS3*.

(a) Panicles, panicle length, grains, and grain length in WT (wild type ZH11, n = 7), *GS3-1Ri* (n = 14), *dep1OE* (n = 10), and *GS3-1Ri/dep1OE* (n = 28) plants. Values are given as mean ± SEM. Different letters indicate significant differences ranked by the LSD test ($P < 0.05$). Panicle, bar = 5 cm; grains, bar = 5 mm.

(b) Description for the T-DNA insertion mutant of *GS3* (*gs3*). The genotype of the mutant is confirmed using PCR. T-DNA is inserted into the second intron. SF28, SR17, and 2715R indicate primers for genotyping. RB and LB indicate the right border and left border of the T-DNA. The transcription level of *GS3* is investigated in 2 cm panicle. W, the wild type Dongjing; H, plants heterozygous for T-DNA insertion; M, plants homozygous for T-DNA insertion.

(c) Grains and grain length of WT (wild type Dongjing) (n = 8), *gs3* (n = 7), *GG*-1 (n = 10), *GG*-2 (n = 10), *DG*-1 (n = 14), *DG*-2 (n = 11), *Gd*-1 (n = 7), *Gd*-2 (n = 12), *Dd*-1 (n = 18), and *Dd*-2 (n = 8). Values are given as mean ± SEM. *P* values are given based on two-tailed *t*-tests. *GG* and *DG* indicate *gs3* plants transformed with *GS3-1* under *GS3* and *DEP1* promoters, respectively. *Gd* and *Dd* indicate *gs3* plants transformed with *dep1* under *GS3* and *DEP1* promoters, respectively. Bar = 5 mm.

(d) Grains and grain length of WT (wild type ZH11) (n = 6), *DEP1*^{ko} (n = 7), *GS3-1OE* (n = 10), *GS3-1OE/DEP1*^{ko} (n = 8), *GS3-4OE* (n = 6), and *GS3-4OE/DEP1*^{ko} (n = 8). Values are given as mean ± SEM. Different letters indicate significant differences ranked by the LSD test ($P < 0.05$). Bar = 5 mm.

(e) Grains and grain length of *GS3-1OE* (n = 5), *GS3-1OE/DEP1OE* (n = 13), *GS3-1Ri* (n = 17), and *GS3-1Ri/DEP1OE* (n = 26). Values are given as mean ± SEM. *P* values are given based on two-tailed *t*-tests. Bar = 5 mm.

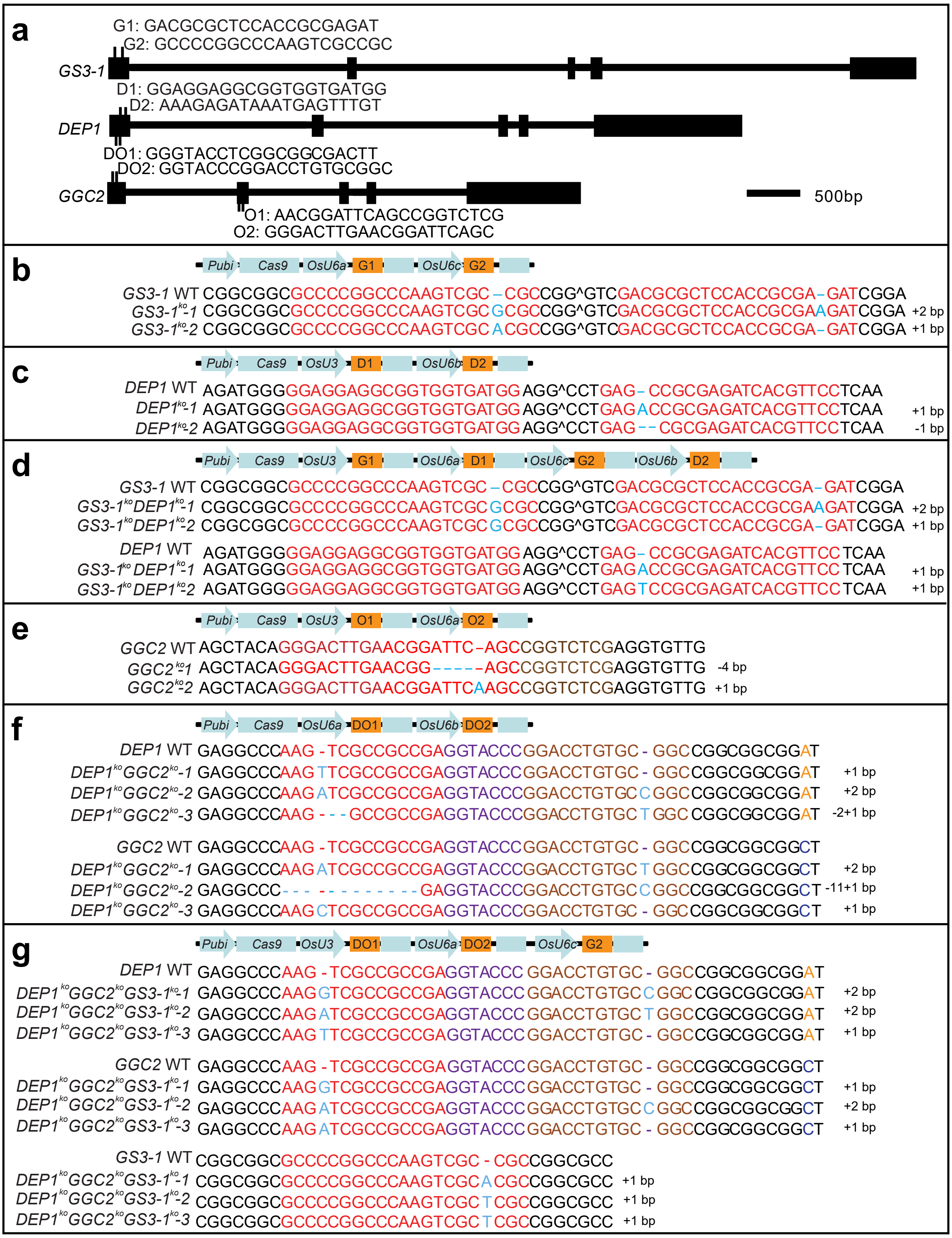

Supplementary Figure 3 Knock-out mutants of three Gγ proteins created by CRISPR/Cas9.
(a) Schematic map of the sgRNA target sites in *GS3*, *DEP1*, and *GGC2*.
(b) Vector construction and sequence alignment for *GS3-1*^{ko} single mutant.
(c) Vector construction and sequence alignment for *DEP1*^{ko} single mutant.
(d) Vector construction and sequence alignment for *GS3-1*^{ko}*DEP1*^{ko} double mutant.
(e) Vector construction and sequence alignment for *GGC2*^{ko} single mutant.
(f) Vector construction and sequence alignment for *DEP1*^{ko}*GGC2*^{ko} double mutant.
(g) Vector construction and sequence alignment for *DEP1*^{ko}*GGC2*^{ko} *GS3-1*^{ko} triple mutant.

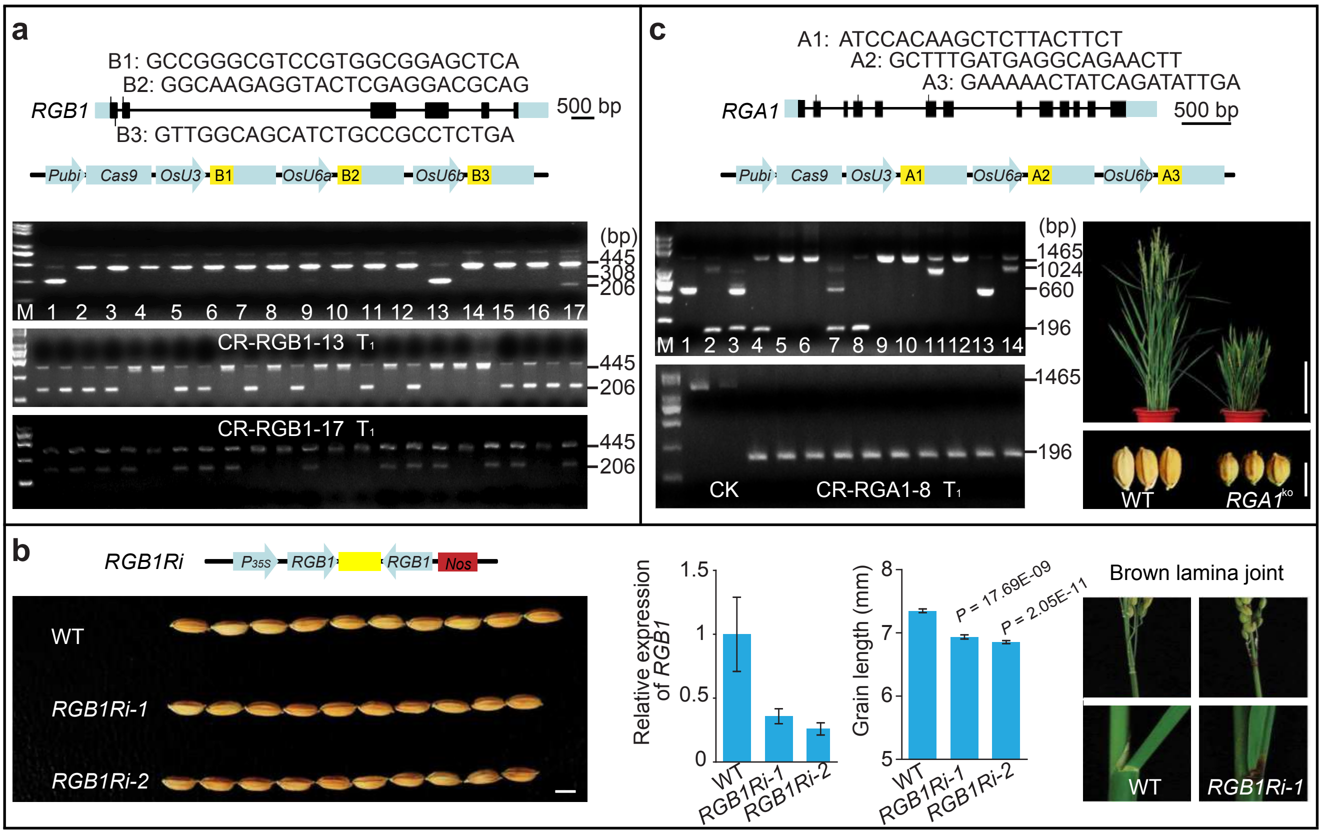

Supplementary Figure 4 Generation of knock-out and knock-down transgenic plants of *RGB1* and *RGA1*.

(a) Generation of *RGB1*^{ko} mutant by CRISPR/Cas9 technology in ZH11 background. The vector and schematic map of the sgRNA target sites in *RGB1* are shown. The genotypes of 17 T_0 transgenic plants and two T_1 families from *RGB1*^{ko}*-13* and *RGB1*^{ko}*-17* were investigated, and no homozygous mutation was identified.

(b) Generation of transgenic plants with suppression of *RGB1* in ZH11 background. The vector construction for *RGB1Ri* is shown. Relative expression level of *RGB1* in *RGB1Ri* transgenic plants is given as mean ± SEM (n = 3). The grain length of WT (wild type ZH11), *RGB1Ri-1*, and *RGB1Ri-2* is given as mean ± SEM (n = 10). *P* values are given based on two-tailed *t*-tests. Bar = 5 mm. Brown lamina joint was observed in *RGB1Ri* transgenic plants.

(c) Generation of *RGA1*^{ko} mutant by CRISPR/Cas9 technology in ZH11 background. The vector and schematic map of the sgRNA target sites in *RGA1* are shown. The genotypes of 14 T_0 transgenic plants and one T_1 family from *RGA1*^{ko} *-8* were investigated. Whole plants and grains of WT (wild type ZH11) and *RGA1*^{ko} mutant are shown. Plant, bar = 20 cm; grains, bar = 5 mm.

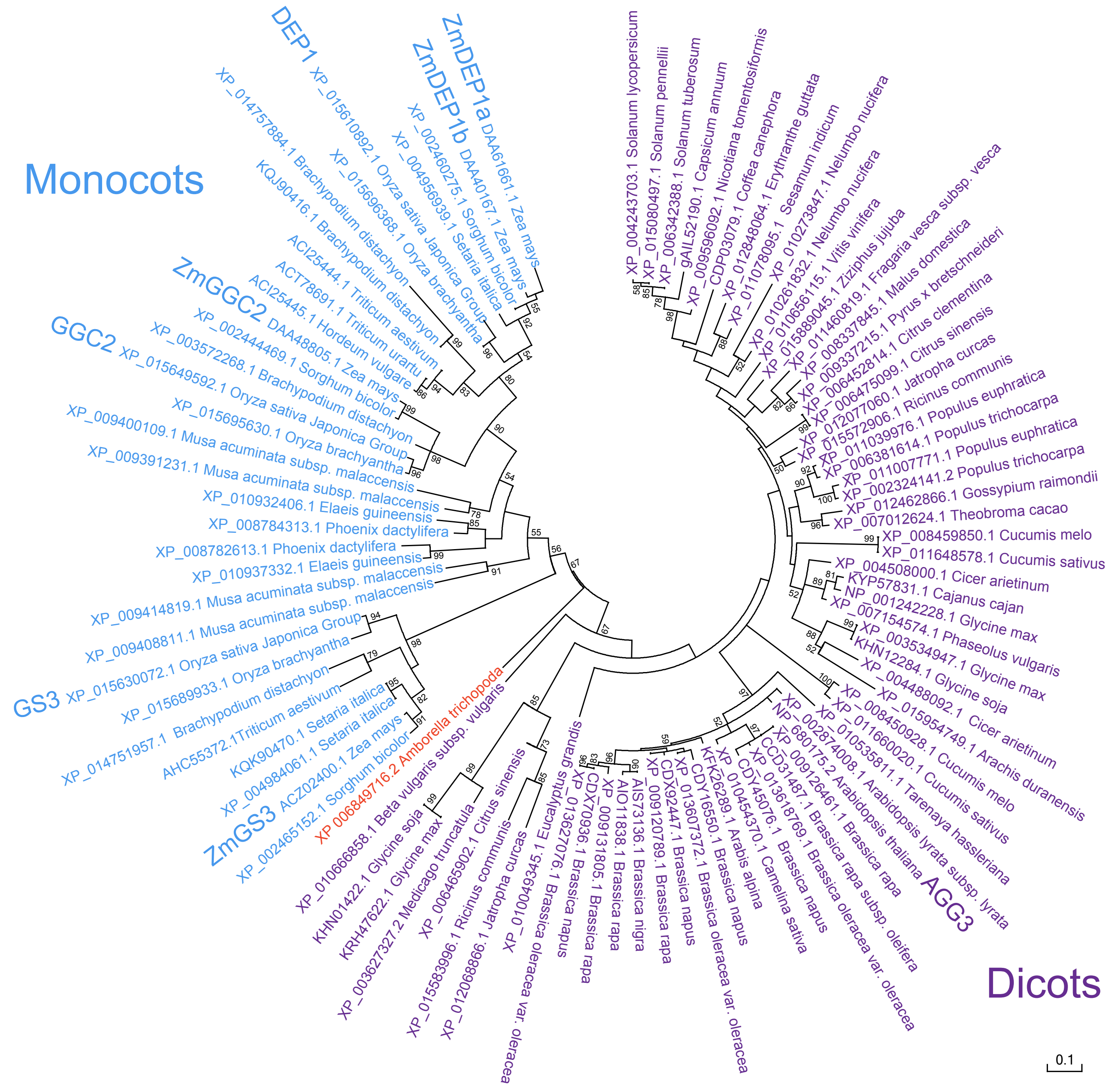

Supplementary Figure 5 The maximum-likelihood tree based on protein sequences of 96 Gγ proteins from 55 species showing significant similarity to three rice Gγ proteins.

The blue color indicates proteins from monocots, and the purple color indicates ones from dicots. One protein from a relict plant is shown in red. The accession number and species for each of the protein is labeled in the tree. Scale bar represents 0.1 substitutions per site.

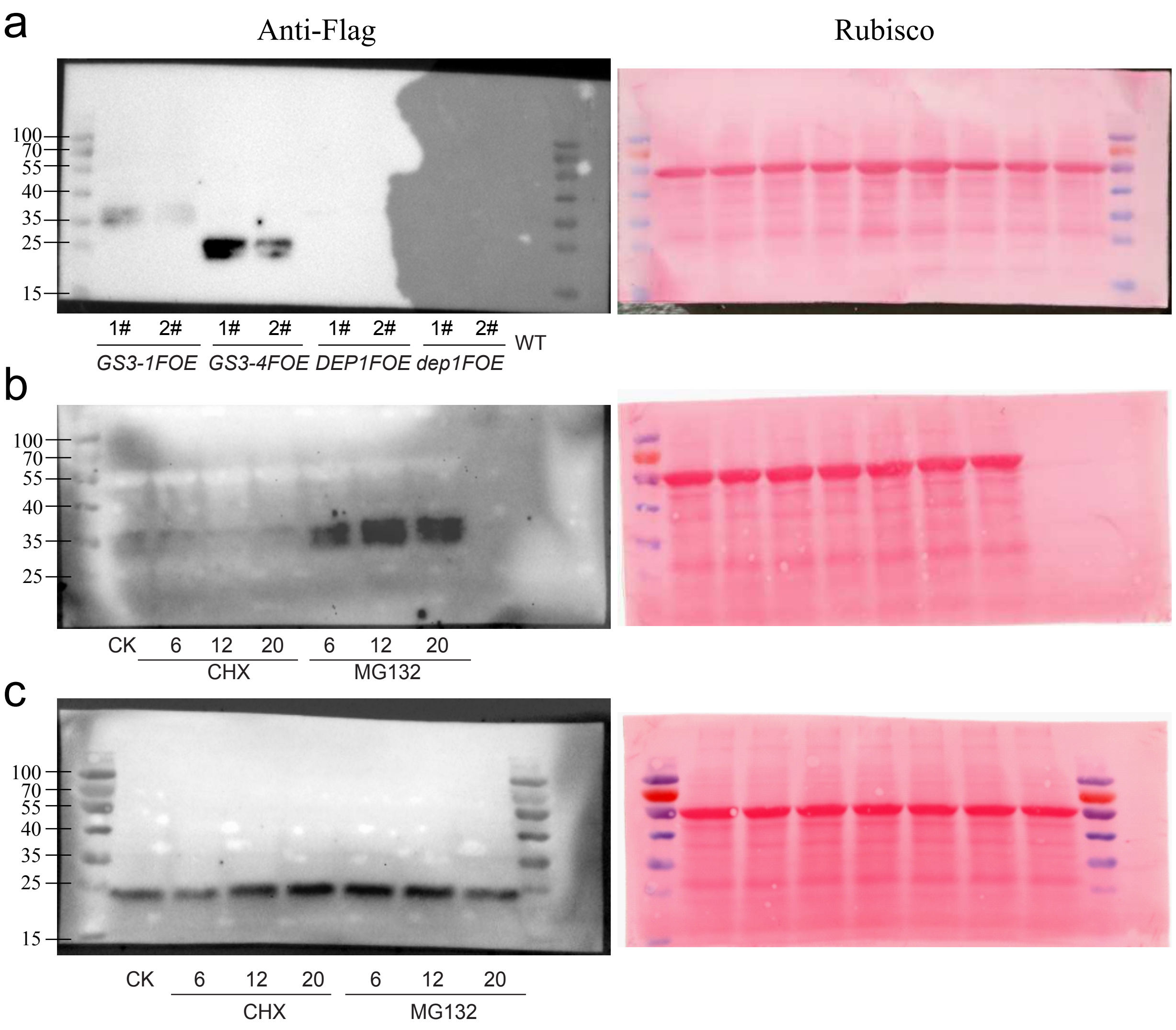

Supplementary Figure 6 Immunoblot analysis of the proteins in transgenic plants with Flag tag.

(a) Immunoblot analysis of the transgenic plants. Total proteins are extracted from the shoots of 15-day-old seeding, and 20 µg amount was used for western blotting.

(b-c) Immunoblot analysis of Flag fused GS3-1(b) and GS3-4 (c) proteins after the treatments with 30 µM CHX and 50 µM MG132. CK, the *GS3-1F* and *GS3-4F* transgenic plants without treatment. CHX, Actidione. The two bands of GS3-1 protein are likely due to protein modification by ubiquitination. The numbers (6, 12, 20) indicate hours after the treatments.

Supplementary Table 1 Phenotypes of transgene positive and negative plants in the T_1 or T_2 generation.

Genotype	Number of plants	Grain length	Number of plants	Grains per panicle
DEP1OE-1 **(+)**	14	7.95 ± 0.04	10	183.80 ± 2.52
DEP1OE-1 **(-)**	12	7.44 ± 0.03	9	200.33 ± 5.80
**Variation**		6.85%		-8.25%
P **-value**		1.32E-10		2.42E-02
DEP1OE-2 **(+)**	18	7.89 ± 0.04	9	173.67 ± 4.63
DEP1OE-2 **(-)**	8	7.20 ± 0.05	8	213.63 ± 12.31
**Variation**		9.58%		-18.71%
P **-value**		1.10E-08		1.41E-02
DEP1OE-3 **(+)**	20	7.93 ± 0.04	13	192.69 ± 5.39
DEP1OE-3 **(-)**	10	7.30 ± 0.03	10	218.40 ± 9.11
**Variation**		8.63%		-11.77%
P **-value**		4.60E-12		0.0281
dep1OE-1 **(+)**	16	6.92 ± 0.03	14	233.79 ± 9.05
dep1OE-1 **(-)**	4	7.26 ± 0.01	12	225.17 ± 10.03
**Variation**		-4.68%		3.83%
P **-value**		7.12E-10		0.53
dep1OE-2 **(+)**	17	6.75 ± 0.04	11	190.30 ± 10.78
dep1OE-2 **(-)**	11	7.07 ± 0.03	11	183.55 ± 9.23
**Variation**		-4.53%		3.68%
P **-value**		1.47E-07		0.64
DEP1Ri-1 **(+)**	23	6.69 ± 0.03	10	211.20 ± 3.47
DEP1Ri-1 **(-)**	7	7.10 ± 0.05	10	221.70 ± 5.19
**Variation**		-5.77%		-4.74%
P **-value**		3.35E-05		0.11
DEP1Ri-2 **(+)**	12	6.65 ± 0.05	10	215.00 ± 3.46
DEP1Ri-2 **(-)**	5	7.19 ± 0.03	10	215.6 ± 4.61
**Variation**		-7.51%		-0.28%
P **-value**		1.04E-07		0.92
GS3-1OE-1 **(+)**	12	6.73 ± 0.05	15	179.93 ± 8.41
GS3-1OE-1 **(-)**	7	7.33 ± 0.03	14	210.71 ± 6.97
**Variation**		-8.19%		-14.61%
P **-value**		6.56E-09		9.03E-03
GS3-1OE-2 **(+)**	9	6.41 ± 0.08	15	189.80 ± 4.24
GS3-1OE-2 **(-)**	6	7.19 ± 0.06	13	202.15 ± 6.60
**Variation**		-10.85%		-6.11%
P **-value**		2.50E-06		0.13
GS3-1OE-3 **(+)**	9	6.97 ± 0.03	15	188.40 ± 6.43
GS3-1OE-3 **(-)**	8	7.59 ± 0.06	13	198.69 ± 5.90
**Variation**		-8.17%		-5.18%
P **-value**		2.11E-06		0.25
GS3-4OE-1 **(+)**	13	5.91 ± 0.06	11	251.55 ± 11.35
GS3-4OE-1 **(-)**	6	7.21 ± 0.05	8	233.13 ± 11.83

**Variation**		-18.03%		7.90%
***P*-value**		2.57E-11		0.28
GS3-4OE-2 **(+)**	12	5.91 ± 0.05	9	253.67 ± 10.27
GS3-4OE-2 **(-)**	5	7.21 ± 0.08	8	215.00 ± 11.55
**Variation**		-18.03%		17.99%
***P*-value**		2.82E-06		0.02
GS3-4OE-3 **(+)**	10	5.67 ± 0.08	8	245.63 ± 8.80
GS3-4OE-3 **(-)**	6	7.20 ± 0.07	8	233.75 ± 10.00
**Variation**		-21.25%		5.08%
***P*-value**		1.63E-09		0.12
GS3Ri-1 **(+)**	18	7.83 ± 0.02	14	181.93 ± 2.99
GS3Ri-1 **(-)**	12	7.33 ± 0.04	11	205.64 ± 2.22
**Variation**		6.82%		-11.53%
***P*-value**		6.16E-10		1.92E-06
GS3Ri-2 **(+)**	19	7.46 ± 0.03	11	208.73 ± 6.22
GS3Ri-2 **(-)**	11	7.09 ± 0.03	14	245.17 ± 8.06
**Variation**		5.22%		-14.86%
***P*-value**		2.51E-09		1.15E-03
GS3Ri-3 **(+)**	26	7.76 ± 0.02	13	204.77 ± 1.78
GS3Ri-3 **(-)**	4	7.37 ± 0.04	10	230.30 ± 2.04
**Variation**		5.29%		-11.09%
***P*-value**		2.16E-04		1.11E-08
GGC2OE-1 **(+)**	5	8.13 ± 0.08	12	148.83 ± 1.80
GGC2OE-1 **(-)**	5	7.26 ± 0.01	12	187.50 ± 1.87
**Variation**		11.98%		-20.62%
***P*-value**		9.87E-05		9.34E-14
GGC2OE-2 **(+)**	5	8.07 ± 0.12	12	116.67 ± 1.32
GGC2OE-2 **(-)**	5	7.14 ± 0.03	12	197.92 ± 2.17
**Variation**		13.03%		-41.05%
***P*-value**		1.11E-03		1.94E-17

All data are given as mean ± SEM. *P* values are obtained by Student's *t* tests between the positive and negative plants in the same family.

Supplementary Table 2 Grain length of transgene-positive and transgene-negative segregants in T_1 families in DHX background.

Constructs	Positive (mm)	Negative (mm)	*P*	Grain size variation[a]
DEP1OE	9.01 ± 0.13 (n = 9)	8.11 ± 0.05 (n = 6)	9.19E-05	11.09%
AGG3OE	6.40 ± 0.06 (n = 10)	7.94 ± 0.23 (n = 6)	7.93E-04	-19.31%
AGG3^{OSR}OE	5.22 ± 0.06 (n = 25)	8.42 ± 0.06 (n = 20)	1.34E-33	-38.03%
ZmGS3OE	6.51 ± 0.10 (n = 10)	8.10 ± 0.04 (n = 6)	8.62E-09	-19.61%
ZmGS3^{OSR}OE	5.07 ± 0.05 (n = 8)	8.34 ± 0.09 (n = 6)	1.24E-09	-39.20%
GmDEP1OE	5.61 ± 0.05 (n = 26)	8.61 ± 0.09 (n = 8)	1.59E-12	-34.84%
GS3-1OE	7.32 ± 0.06 (n = 10)	8.24 ± 0.05 (n = 12)	1.83E-10	-11.15%
GS3-4OE	5.44 ± 0.03 (n = 10)	8.05 ± 0.08 (n = 12)	1.71E-14	-32.43%

[a]Extent of grain length variation of the transgene positive plants relative to the negative plants.

[b]All data are given as mean ± SEM. *P* values are obtained by Student's *t* tests between the positive and negative plants in the same family.
