# Supplementary material for: A G-protein pathway determines grain size in rice
Source: Nat Commun. 2018 Feb 27;9:851. doi: 10.1038/s41467-018-03141-y (PMC5829277; doi:10.1038/s41467-018-03141-y)
Supplement: Supplementary file 2 — Description of Additional Supplementary Files [file 41467_2018_3141_MOESM2_ESM.pdf]

Descriptions of Additional Supplementary Files

File Name: Supplementary Dataset 1
Description: Homologs of three Gγ proteins in rice and other species

File Name: Supplementary Dataset 2
Description: Primers used in this study
